# The efficacy of family treatments for adolescent anorexia nervosa in specialist versus non-specialist settings: protocol for a systematic review and meta-analysis

**DOI:** 10.1186/s40337-022-00645-3

**Published:** 2022-08-15

**Authors:** Ashlea Hambleton, Phillip Aouad, Jane Miskovic-Wheatley, Daniel Le Grange, Stephen Touyz, Sarah Maguire

**Affiliations:** 1 InsideOut Institute, The University of Sydney, Sydney, Australia; 2 Sydney Local Health District, Camperdown, Australia; 3 University of California, San Francisco, San Francisco, California USA

**Keywords:** Anorexia nervosa, Family-based treatment, Family therapy, Efficacy, Specialist, Non-specialist

## Abstract

**Background:**

Anorexia nervosa (AN) is often diagnosed in adolescence, and most evidence-based treatments for AN in young people involve the family. Family therapies for AN are intensive, outpatient treatments that utilise the parents as the primary resource in the young person’s recovery. Research regarding family treatment for AN is often conducted in specialist settings—with relatively little data reporting the translation of this specialised treatment into real-world, non-specialist settings. This systematic review and meta-analysis aims to determine the efficacy of family treatments for adolescents with AN in specialist settings versus non-specialist settings.

**Methods:**

This systematic review and meta-analysis will be conducted according to the preferred reporting items for systematic reviews and meta-analyses guidelines. Retrospective cohort studies, pilot studies, case series, randomised controlled trials and qualitative investigations that present original data and investigated the efficacy of family treatments for adolescents with AN in either a specialist or non-specialist setting will be included in the review. Data will be extracted by two reviewers and study quality will be assessed. The primary outcome, change in weight, will be used to determine via meta-analysis and, depending on study heterogeneity, subgroup analysis or meta-regression whether there is a statistically significant subgroup difference between specialist and non-specialist treatment settings. The review will also consider changes in eating disorder symptomology and related constructs.

**Discussion:**

Results from this review will help determine if there is a difference in the efficacy of family treatments for adolescent AN in specialist versus non-specialist treatment settings, primarily in relation to weight recovery. This, in turn, will inform the translation of evidence-based interventions that are generally studied and implemented within specialist centres into the non-specialist health care system.

**Supplementary Information:**

The online version contains supplementary material available at 10.1186/s40337-022-00645-3.

## Background

Characterised by an excessive preoccupation with weight and shape, food restriction, weight loss, and malnutrition, anorexia nervosa (AN) is a severe illness with numerous physical and psychological health impacts [1]. Although observed across the entire lifespan, AN is often first diagnosed during adolescence with an average duration illness of 5–7 years [2]. AN holds the highest mortality rate of all psychiatric conditions; a consequence of a heightened rate of suicide and medical complications [3, 4].

Concerningly, young people are particularly susceptible to the physical impacts of the illness, with complications from malnutrition and compensatory behaviours which includes growth retardation, osteoporosis, amenorrhea, and changes in brain structure [5]. Given the seriousness of the illness, treatment must be delivered efficiently and effectively. Although effective treatment of AN in young people remains an area of clinical and research need, the consensus remains that treatment is most effective when including the family. Several models, including a manualised form known as family-based treatment (FBT) [6] or family therapy for AN (FT-AN) (sometimes referred to as Maudsley Family Therapy (MFT)) [7, 8] have garnered an evidence base as the recommended treatment for adolescent AN [5, 9].

FT (herein encompassing FBT, FT-AN and MFT) is an intensive, outpatient treatment that utilises the family as the primary resource towards the goal of getting the young person with AN well. The initial focus of treatment is weight restoration and disrupting ED behaviours via the parents/guardians. As weight restoration and behavioural symptom resolution are facilitated, less parental authority is typically required, and parents may gradually restore autonomy over eating back to the adolescent. Remission rates are particularly promising for adolescents with short illness duration [10–12]. The average remission rate for all participants at the end of FBT is approximately 50% [10, 13–15], and 75% of young people demonstrate improvement in weight and eating-related symptomatology at the end of FBT [8, 11]. Few studies have explored the long-term outcomes for FT; however preliminary evidence suggests that treatment effects are maintained for up to 5 years [16, 17]. However further replication in larger samples is needed.

The efficacy of FT so far has been examined in randomised controlled trials (RCTs) and systematic reviews [10]; however, these results are constrained to specialised settings such as university research units or specialist hospitals. Indeed, most research regarding the efficacy of psychological and behavioural treatments for AN (across the lifespan) has taken place within specialist, research-led settings [18], and so generalisability to those not included in trial eligibility criteria is limited. In an effort to bridge the research-practice gap, a growing body of literature (reports and case series) has reported the effectiveness outcomes of FT in non-specialist settings (i.e., digitally, in clinical practice, general practice, and community health settings) [19–22]. However, these results have not been synthesised systematically. This lack of translation to ‘real-world’ prevents a fair evaluation of the effectiveness of FT for AN in non-specialist settings, which is necessary, given this is where much of the population accesses treatment [18, 23].

This systematic review will gather and organise evidence from a diverse range of studies to critically evaluate the efficacy of FT in specialist compared to non-specialist settings. The current protocol outlines the methods of our investigation, in accordance with the PRISMA checklist [24]. This systematic review's primary aim is to determine if there is a difference in the efficacy of FT for AN in specialist versus non-specialist treatment settings. A secondary aim is to evaluate the methodological quality of included studies. We hope that findings from this review will inform the translation of evidence-based interventions that are generally studied and implemented within specialist centres into the non-specialist health care system. Further, it is anticipated that the outcomes derived from non-specialist settings may inform the future review and development of specialist protocols, that is, what can be learned from how these treatments are implemented in the ‘real world’.

## Methods

### Eligibility criteria

The systematic review and meta-analysis will be conducted according to the Preferred Reporting Items for Systematic Reviews and Meta-Analyses (PRISMA) guidelines, and studies will be selected for inclusion in this review according to the criteria outlined below.

#### Study designs

Retrospective cohort studies, pilot studies, case series, randomised controlled trials and qualitative investigations that present original data and investigated the efficacy of FT for AN in either a specialist or non-specialist setting will be eligible for inclusion in the review. If data are reported for the same intervention across multiple studies, for example, a pilot study and then a subsequent randomised controlled trial, only data from the randomised controlled trial will be extracted for analysis. Select ‘grey literature’ (e.g., non-examined or non-peer reviewed works; non-government or professional association works), theoretical or conceptual articles and conference abstracts will be excluded.

#### Participants

Studies that included young people, defined as participants aged up to 21 years (inclusive) with a primary diagnosis of AN or Atypical AN (AAN) will be included.

#### Interventions

Eligible studies must include eating disorder focussed FT (including FT-AN, MFT, FBT or Multiple FBT) for AN or AAN as the treatment under investigation.

#### Comparators

Treatment setting, specifically the outcomes of FT for AN/AAN in specialist versus non-specialist settings, will be compared.

#### Outcomes

*Primary Outcome*: The key goal of FT for adolescents with a restricting eating disorder is the restoration and normalisation of weight (i.e., change in weight), and will be defined as the primary outcome measure for this analysis. Sufficient data are required to calculate effect sizes (e.g., M, SD, and/or, n at pre-treatment, post-treatment or follow up). Thus, a pre-treatment and at least one post-treatment or follow-up assessment are necessary.

*Secondary Outcomes*: Changes in associated eating disorder psychopathology and non-eating disorder psychopathology will be reviewed as secondary outcomes, should there be sufficient data. Examples of likely secondary outcome variables include changes in global eating disorder symptomology, restriction, compensatory behaviours (exercise, purging), shape and/or weight (body image) concerns, as well as changes in depression and anxiety/distress severity, quality of life and daily functioning.

#### Setting

Studies must have taken place within a specialist or non-specialist setting.

Specialist settings are defined as those where studies are run within clinical or research settings specialised in the treatment of AN; for example, a university eating disorder research centre (such as those seen at Stanford University, University of California San Francisco or The University of Chicago) as well as specialist eating disorder treatment hospitals or centres (such as the Eating Disorder Program Westmead Children’s Hospital, Sydney; The Royal Children’s Hospital Eating Disorders Service, Melbourne; or the Eating Disorder Service Maudsley Hospital, London). Essentially, a specialist setting is one that has been developed solely for the research and treatment of AN.

Non-specialist settings are defined as those where FT has been delivered in community health settings, online/telehealth or private practices. While FT may have been delivered by specialist clinicians trained in FT, the setting itself is not a specialist research/clinical centre.

#### Covariates

Anticipated covariates that will need to be controlled include (but may not be limited to); frequency of sessions, duration of treatment, duration of illness, presence of comorbidities, length of follow up, and if applicable, treatment fidelity. An umbrella term for FT is being used to capture studies that examine the effectiveness of FT-AN and FBT. While there may be some nuance differences in the treatment approaches, they share common treatment principles (family as the primary resource to support and direct refeeding, externalisation of the illness, recovery is possible, carers are not to blame for the illness). However, the authors will pay particular attention to details regarding the fidelity to the treatment model used, and any studies that deviate significantly from the treatment manual will be highlighted and limitations of these studies will be discussed in greater detail.

#### Language

No limitation on language will exist. However, a translation into English will be sought for studies that meet inclusion criteria. This will be conducted with credible services or utilise academics fluent in the relevant language.

### Data collection and analysis

#### Search strategy and study selection

A broad search strategy (see Additional File 1) will be used across seven databases (from database inception until July 2022): (1) PsycInfo of the American Psychological Association, (2) MEDLINE/PubMed, (3) Embase, (4) Web of Science, (5) Cochrane Library, (6) CINHAL, and (7) Scopus. The reference lists of included studies will be manually searched to find additional eligible studies. Studies citing relevant articles will also be reviewed using the ‘cited by’ function in Google Scholar.

Literature management will be conducted using Covidence [25]. Following removal of duplicates, the titles and abstracts of studies retrieved using the database searches will be screened by two reviewers. The full text of potentially eligible studies will be retrieved and assessed for inclusion in the review by two reviewers (AH and PA). Should the full text of a study not be accessible through institutional memberships, study authors will be contacted to retrieve the manuscript. The decision to include studies will be based on criteria outlined in the preceding section. A third reviewer will resolve discrepancies between the first two reviewers at both stages. These two reviewers must also approve the inclusion of further studies identified from reference lists of the eligible articles found using database searches. The reason for exclusion of any study will be recorded, and the study selection process will be presented in a PRISMA flow diagram.

#### Data extraction

Data extraction will be performed independently by two researchers. One researcher (AH) will extract all required study data, and the second researcher (PA) will cross-check the data extraction to ensure integrity and quality are maintained and that the information can be used in meta-analytic comparisons and risk of bias assessment [26].

Data collection refers to: publication details; study information including design, inclusion and exclusion criteria, assessment time points, sample size; intervention information (if applicable) including comparison groups or controls, setting, duration, and compliance; participant information (population/demographics, exposure/illness information, comorbidities) and study results including primary and secondary outcomes, statistical methods and adjusted and non-adjusted data. Data will be recorded for pre-treatment, post-treatment and/or follow-up, where available. Where data are missing for the metanalysis, we will attempt to contact the study authors to obtain this.

#### Quality assessment (risk of bias)

The methodological quality of included studies will be appraised using the Ferro and Speechley (2009) quality checklist, a modified version of the Downs and Black index. (27) After data extraction, one author (AH) will independently score all included studies, and a second author (PA) will screen a 50% random selection of eligible articles. Inter-rater agreement will be calculated using the Interclass Correlation statistic. Large discrepancies in quality evaluation will be resolved through discussion with the third reviewer.

The Cochrane Collaboration's Risk of Bias Tool [26] will be used to assess the risk of bias in randomised-controlled studies as well as non-randomised studies. Summary assessments of low, medium or high risk of bias will be performed as recommended [26]. The risk of bias will be examined during data extraction, following the procedure of independent coding and disagreement resolution described above, and will be used to interpret outcome data.

### Meta-analytic approach

#### Meta-analysis

Before examining relationships between intervention setting and outcomes, overall effect sizes will be calculated for changes from pre-treatment to post-treatment and from pre-treatment to follow up when available. Effects will be calculated based on means, standard deviations (SDs), and sample sizes reported within studies. If this data is unavailable, p- or t-values with sample means will be used instead.

#### Subgroup analyses/meta-regression

Depending on the heterogeneity of studies, a subgroup analysis or meta-regression will be performed to determine whether there is a statistically significant subgroup difference in treatment effectiveness, defined as change in weight, between specialist and non-specialist treatment settings. To do this, we will test the difference between the pooled effect estimates for each subgroup [28]. Heterogeneity will be determined by a Cochran’s Q test and a Higgins’s I2 statistic [26]. Plausibility and significance of interaction (between subgroups), covariate distribution (the number of trials and participants contributing to each subgroup), and potential confounding factors will also be considered during analysis [28].

### Ethics and dissemination

No human subject participants will be involved. On completion of the analysis, we will prepare a manuscript for publication in a peer-reviewed journal and present the results at conferences.

## Discussion

This systematic review aims to determine and identify any differences in the efficacy of FT for AN in specialist versus non-specialist settings. Much of the existing peer-reviewed evidence base for the treatment of AN has emerged from RCTs within specialised clinical research settings. However, individuals with AN and their families typically seek treatment from local community settings, often disconnected from specialist multi-disciplinary teams. Further, many young people presenting to these non-specialist services also experience significant comorbidities, such as suicidal ideation, self-harm or psychotic symptoms, that may have meant they were excluded from clinical trials [29]. Thus, it is worthwhile to determine the efficacy of FT for this ‘real-world’ population group.

This method has several limitations that should be considered during data collection, analysis and interpretation. To capture the scope of work that has been completed regarding the specialist family treatment of adolescent AN, we have utilised broad eligibility criteria. However, this will also introduce numerous confounding variables that must be considered and managed. There are several strengths to this method. We will include a broad range of study designs to capture non-specialist settings and provide a complete overview of the literature. Secondly, we will conduct a thorough quality assessment of all included studies using an established assessment methodology. To facilitate transparency, the full search strategy that will be used to find relevant studies has been made available, and all data extracted from included studies will be published with the final manuscript.

This systematic review will provide a detailed summary of the effectiveness of FT for adolescent AN in specialist and non-specialist settings. The meta-analysis will further detail the effectiveness outcomes, and what, if any, differences because of treatment setting ought to be considered. We hope that findings from this review will inform the research-treatment gap, with learnings regarding: (1) how effective are the treatments tested within labs and specialist settings when they are implemented into the very population they were designed to treat (the ‘real world’), and, (2) how future treatment protocols can be informed by the outcomes from non-specialist settings.

## Supplementary Information

**Additional file 1:** Systematic Review Search Strategy.

## Data Availability

Not applicable—all citations provided.

## Abbreviations

AN: Anorexia nervosa
AAN: Atypical anorexia nervosa
ED: Eating disorder
FBT: Family based treatment
FT-AN: Family therapy for anorexia nervosa
MFT: Maudsley family therapy
PRISMA: Preferred reporting items for systematic reviews and meta-analyses
RCT: Randomised controlled trial
