# Supplementary material for: The efficacy of family treatments for adolescent anorexia nervosa in specialist versus non-specialist settings: protocol for a systematic review and meta-analysis
Source: J Eat Disord. 2022 Aug 15;10:120. doi: 10.1186/s40337-022-00645-3 (PMC9380342; doi:10.1186/s40337-022-00645-3)
Supplement: Supplementary file 1 — Additional file 1: Systematic Review Search Strategy. [file 40337_2022_645_MOESM1_ESM.docx]

**Supplementary File 1**

**Search Strategy**

**Words relating to Anorexia Nervosa**

Anorex*
Feeding and eating disorder*

Eating Disord*

Restrict*

**Words relating to Treatment**

Family therap*

Family based treatment or family-based treatment

Family based therapy

FBT

Maudsley

**Words relating to Young People**

Adolesc*

Young adult

Young pe*

Youth

Teen*

Child*

(“Family therapy” OR family therap* OR “family based treatment” OR “family-based therapy” OR “family based” OR “family-based” OR FBT OR Maudsley*) AND (Anorex* OR Feeding and Eating Disorder OR Eating Disord* OR Restrict*) AND (Adolesc* OR young adult OR young pe* OR youth OR teen* OR Child*)
